# Impact of health literacy on anxiety and depressive symptoms in pregnant women in Japan during the COVID-19 pandemic

**DOI:** 10.1038/s41598-022-18405-3

**Published:** 2022-08-18

**Authors:** Yasuo Haruyama, Etsuko Miyagi, Gen Kobashi, Soichiro Obata, Takeshi Umazume, Asuka Yoshimi, Akitoyo Hishimoto, Kentaro Kurasawa, Yukio Suzuki, Tomoaki Ikeda, Tadashi Kimura, Hideto Yamada

**Affiliations:** 1 Integrated Research Faculty for Advanced Medical Sciences, Dokkyo Medical University, 880 Kitakobayashi, Mibu, Tochigi 321-0293 Japan; 2 Department of Obstetrics and Gynecology, Yokohama City University Hospital, Yokohama City University Graduate School of Medicine, 3-9, Fukuura, Kanazawa-ku, Yokohama, Kanagawa 236-0004 Japan; 3 Department of Public Health, Dokkyo Medical University, School of Medicine, 880 Kitakobayashi, Mibu, Tochigi 321-0293 Japan; 4 Perinatal Center for Maternity and Neonates, Yokohama City University Medical Center, 3-9, Fukuura, Kanazawa-ku, Yokohama, Kanagawa 236-0004 Japan; 5 Department of Obstetrics and Gynecology, Hokkaido University Graduate School of Medicine, N15W7, Kita-ku, Sapporo, Hokkaido 060-8648 Japan; 6 Department of Psychiatry, Yokohama City University Hospital, 3-9, Fukuura, Kanazawa-ku, Yokohama, Kanagawa 236-0004 Japan; 7 Obstetrics and Gynecology, Mie University Graduate School of Medicine, 2-174, Edobashi, Tsu, Mie 514-8507 Japan; 8 Department of Obstetrics and Gynecology, Osaka University Graduate School of Medicine, 2-2 Yamadaoka, Suita, Osaka 565-0871 Japan; 9 Department of Obstetrics and Gynecology, Kobe University Graduate School of Medicine, 7-5-1, Kusunoki-cho, Chuo-ku, Kobe, Hyogo 650-0017 Japan

**Keywords:** Anxiety, Depression, Preventive medicine

## Abstract

To investigate the relationships between communicative and critical health literacy (CCHL) and anxiety and depressive symptoms (ADs) in pregnant women during the coronavirus disease 2019 (COVID-19) pandemic. A cross-sectional study was conducted and 5466 pregnant women responded in Japan in September 2020. A Kessler 6 scale (K6) score ≥ 10, an Edinburgh Postnatal Depression Scale (EPDS) score ≥ 13, and four CCHL groups were analyzed using a logistic regression model and trend test. The proportions of pregnant women with a K6 score ≥ 10 and EPDS score ≥ 13 were 13.5 and 15.4%, respectively. In comparisons with the low CCHL group, the adjusted odds ratio (95% CI) for anxiety symptoms was 0.770 (0.604–0.982) in the high CCHL group, while those for depressive symptoms were 0.777 (0.639–0.946), 0.665 (0.537–0.824), and 0.666 (0.529–0.838) in the lower, higher, and high CCHL groups (all p < 0.05), respectively, after adjustments for potential confounding factors, such as age, weeks of gestation, complications, history, number of children, marital status, education, employment, and income. Higher CCHL was associated with significantly lower adjusted odds ratios for anxiety (p for trend = 0.019) and depressive symptoms (p for trend < 0.001). These results suggest a relationship between CCHL and ADs in pregnant women during the COVID-19 pandemic.

## Introduction

In 2020, a worldwide pandemic of novel coronavirus disease 2019 (COVID-19) emerged from Wuhan. Due to mandatory changes in lifestyles under the declaration of a lockdown or emergency state, greater mental health difficulties have been reported in general populations^1–3^.

After the COVID-19 pandemic, the prevalence of major depressive symptoms with an Edinburgh postnatal depression scale (EPDS) score ≥ 13 was 15% for pregnant women in Europe countries, while that of a generalized anxiety symptom score ≥ 10 was 11%^4^. The prevalence of depression with an EPDS score ≥ 10 was 16.1% in Wuhan, China, and that of anxiety with a self-rating anxiety scale score ≥ 50 was 13.9%^5^. In a national survey conducted in Mexico, 17.5% of participants had an EPDS score of more than 14 points^6^. In Japan, the prevalence of depression with an EPDS score ≥ 10 was reportedly 35.5% in pregnant women, while that of anxiety with a Kessler 6 scale (K6) score ≥ 5 was 39.9%^7^. A meta-analysis revealed that the pooled prevalence was 18.7% for anxiety and 25.1% for depression^8^. Although these studies were conducted in various countries and used different cut-off points for EDPS as well as different anxiety scales, anxiety and depressive symptoms were commonly reported in pregnant women after the COVID-19 pandemic.

Since the COVID-19 pandemic, some studies have suggested the urgent need to screen and treat mental health conditions^9,10^ and provide obstetric counseling and psychological support^7,10^ for pregnant women with high anxiety and depression. Health literacy is a personal ability to find, assess, and use health information. Previous studies reported the importance of ensuring a correct understanding of health information and relevant behavior at the onset of novel infectious diseases^11–14^. Although a study reported health literacy, anxiety, and depression among pregnant women^15^, the impact of health literacy for COVID-19 on anxiety and depressive symptoms in pregnant women remains unclear. We hypothesized that health literacy may be related to anxiety and depressive symptoms and that an approach to health literacy that reduces these symptoms needs to be proposed. Therefore, the aim of the present study was to the clarify relationships between anxiety and depressive symptoms and communicative and critical health literacy (CCHL) in pregnant women during the COVID-19 pandemic.

## Results

A total of 5739 pregnant women answered the online survey, and 5466 (95.2%) were analyzed after the exclusion of those with missing values (age = 7, expected delivery date = 5, CCHL = 81, K6 = 167, and EPDS = 13).

The characteristics and backgrounds of participants are shown in Table 1. The proportions of pregnant women in their 30 s, after 28 weeks of gestation, and pregnant for the first time were 63.9, 56.6, and 64.7%, respectively. Pregnant women with complications were those with threatened premature delivery (7.8%), gestational diabetes mellitus (4.2%), placental malposition (1.7%), and multiple pregnancies (1.4%), respectively. The proportion of participants with fetal disorder or fetal growth restriction, gestational hypertension, and other complications were less than 1%. Pregnant women with a medical history of 1% or more included miscarriage (19.4%), mental disease (4.3%), premature birth (1.9%), and fatality (1.5%). A total of 94.5% of pregnant women were married and living together, 57.6% were educated beyond university level, 19.5% were unemployed, and 46.0% had an annual household income of less than 7 million (Yen). Mean (SD) CCHL, K6, and EPDS were 17.5 (3.6), 4.6 (4.4), and 7.0 (5.5), respectively.

**Table 1:** Characteristics of pregnant woman (n = 5466).

	n	%
**Age group**		
≤ 19 yr	14	0.3
20–29 yr	1608	29.4
30–39 yr	3493	63.9
40–49 yr	351	6.4
**Weeks of gestation**		
Early pregnancy (≤ 15 wk)	851	15.6
Mid-pregnancy (16–27 wk)	1520	27.8
Late pregnancy (≥ 28 wk)	3095	56.6
**Number of children born**		
0	3535	64.7
1	1388	25.4
2	427	7.8
≥ 3	101	1.8
Unknown	15	0.3
**Complications during pregnancy**^**a**^**, yes**		
Threatened premature delivery	424	7.8
Fetal disorder or fetal growth restriction	51	0.9
Placental malposition	93	1.7
Multiple pregnancy	76	1.4
Gestational hypertension	29	0.5
Gestational diabetes mellitus	231	4.2
Other	970	17.7
**Medical history**^**a**^		
Miscarriage	1061	19.4
Fatal death	84	1.5
Premature birth	106	1.9
Hypertension	38	0.7
Diabetes mellitus	25	0.5
Mental disease	235	4.3
Other	1098	20.1
**Marital status**		
Married and live together	5165	94.5
Married and separated	159	2.9
Unmarried with a partner	63	1.2
Unmarried without a partner	57	1.0
Other	6	0.1
Unknown	16	0.3
**Education**		
Junior high school	96	1.8
High school	803	14.7
College	1389	25.4
University	2748	50.3
Graduate school	397	7.3
Unknown	33	0.6
**Current employment status**		
Full-time	2330	42.6
Part-time	482	8.8
Housewife or student	1536	28.1
On leave	990	18.1
Unemployed	75	1.4
Unknown	53	1
**Household income, yen**		
< 1 million	34	0.6
1–3.99 million	626	11.5
4–6.99 million	1854	33.9
7–9.99 million	1361	24.9
≥ 10 million	964	17.6
Unknown	627	11.5
K6, mean, SD, score	4.6	4.4
EPDS, mean, SD, score	7.0	5.5
CCHL scale, mean, SD, score	17.5	3.6

*CCHL scale* communicative and critical health literacy scale, *K6* Kessler 6 scale, *EPDS* Edinburgh postnatal depression scale.

^a^Multiple answers.

Among pregnant women, 744 (13.6%) showed anxiety symptoms and 844 (15.4%) had depressive symptoms (Table 2). Anxiety and depressive symptoms (ADs) were related to age, the number of children born, two complications during pregnancy (threatened premature delivery and fetal disorder or fetal growth restriction), a history of mental disease, marital status, education level, employment status, household income, and CCHL, while anxiety symptoms were related to a history of hypertension (Table 2).

**Table 2:** Comparison of anxiety and depressive symptoms for each variable in pregnant woman.

	K6 scores		p value^a^	EPDS scores		p value^a^
	< 10	≥ 10		< 13	≥ 13	
	n (%)	n (%)		n (%)	n (%)	
All	4722 (86.4)	744 (13.6)		4622 (84.6)	844 (15.4)	
**Age group**						
≤ 19 yr	9 (0.2)	5 (0.7)	< 0.001	8 (0.2)	6 (0.7)	< 0.001
20–29 yr	1344 (28.5)	264 (35.5)		1288 (27.9)	320 (37.9)	
30–39 yr	3054 (64.7)	439 (59.0)		3012 (65.2)	481 (57.0)	
40–49 yr	315 (6.7)	36 (4.8)		314 (6.8)	37 (4.4)	
**Weeks of gestation**						
Early pregnancy (≤ 15 wk)	719 (15.2)	132 (17.7)	0.123	705 (15.3)	146 (17.3)	0.202
Mid-pregnancy (16–27 wk)	1307 (27.7)	213 (28.6)		1279 (27.7)	241 (28.6)	
Late pregnancy (≥ 28 wk)	2696 (57.1)	399 (53.6)		2638 (57.1)	457 (54.1)	
**Number of children born**						
0	3029 (64.1)	506 (68.0)	< 0.001	2946 (63.7)	589 (69.8)	< 0.001
1	1218 (25.8)	170 (22.8)		1218 (26.4)	170 (20.1)	
2	386 (8.2)	41 (5.5)		377 (8.2)	50 (5.9)	
≥ 3	79 (1.7)	22 (3.0)		73 (1.6)	28 (3.3)	
Unknown	10 (0.2)	5 (0.7)		8 (0.2)	7 (0.8)	
**Complications during pregnancy, yes**						
Threatened premature delivery	352 (7.5)	72 (9.7)	0.035	323 (7.0)	101 (12.0)	< 0.001
Fetal disorder or fetal growth restriction	38 (0.8)	13 (1.7)	0.013	35 (0.8)	16 (1.9)	0.002
Placental malposition	85 (1.8)	8 (1.1)	0.155	80 (1.7)	13 (1.5)	0.694
Multiple pregnancy	68 (1.4)	8 (1.1)	0.430	59 (1.3)	17 (2.0)	0.092
Gestational hypertension	25 (0.5)	4 (0.5)	0.570^b^	23 (0.5)	6 (0.7)	0.285^b^
Gestational diabetes mellitus	209 (4.4)	22 (3.0)	0.064	196 (4.2)	35 (4.1)	0.901
Other	832 (17.6)	138 (18.5)	0.538	819 (17.7)	151 (17.9)	0.905
**Medical history, yes**						
Miscarriage	927 (19.6)	134 (18.0)	0.299	913 (19.8)	148 (17.5)	0.134
Fatal death	73 (1.5)	11 (1.5)	0.899	70 (1.5)	14 (1.7)	0.754
Premature birth	93 (2.0)	13 (1.7)	0.683	92 (2.0)	14 (1.7)	0.520
Hypertension	34 (0.7)	4 (0.5)	0.578	26 (0.6)	12 (1.4)	0.006
Diabetes mellitus	22 (0.5)	3 (0.4)	1.000^a^	19 (0.4)	6 (0.7)	0.261^b^
Mental disease	137 (2.9)	98 (13.2)	< 0.001	144 (3.1)	91 (10.8)	< 0.001
Other	943 (20.0)	155 (20.8)	0.586	932 (20.2)	166 (19.7)	0.741
**Marital status**						
Married and live together	4486 (95.0)	679 (91.3)	< 0.001	4403 (95.3)	762 (90.3)	< 0.001
Married and separated	133 (2.8)	26 (3.5)		120 (2.6)	39 (4.6)	
Unmarried with a partner	47 (1.0)	16 (2.2)		41 (0.9)	22 (2.6)	
Unmarried without a partner	39 (0.8)	18 (2.4)		43 (0.9)	14 (1.7)	
Other	5 (0.1)	1 (0.1)		5 (0.1)	1 (0.1)	
Unknown	12 (0.3)	4 (0.5)		10 (0.2)	6 (0.7)	
**Education**						
Junior high school	69 (1.5)	27 (3.6)	< 0.001	62 (1.3)	34 (4.0)	< 0.001
High school	670 (14.2)	133 (17.9)		630 (13.6)	173 (20.5)	
College	1199 (25.4)	190 (25.5)		1158 (25.1)	231 (27.4)	
University	2401 (50.8)	347 (46.6)		2383 (51.5)	366 (43.3)	
Graduate school	355 (7.5)	42 (5.6)		361 (7.8)	36 (4.3)	
Unknown	28 (0.6)	5 (0.7)		29 (0.6)	4 (0.5)	
**Current employment status**						
Full-time	2073 (43.9)	257 (34.5)	< 0.001	2021 (43.7)	309 (36.6)	< 0.001
Part-time	419 (8.9)	63 (8.5)		414 (9.0)	68 (8.1)	
Housewife or student	1291 (27.3)	245 (32.9)		1287 (27.8)	249 (29.5)	
On leave	842 (17.8)	148 (19.9)		814 (17.6)	176 (20.9)	
Unemployed	54 (1.1)	21 (2.8)		46 (1.0)	29 (3.4)	
Unknown	43 (0.9)	10 (11.3)		40 (0.9)	13 (1.5)	
**Household income, yen**						
< 1 million	27 (0.6)	7 (0.9)	< 0.001	23 (0.5)	11 (1.3)	< 0.001
1–3.99 million	503 (10.7)	123 (16.5)		474 (10.3)	152 (18.0)	
4–6.99 million	1570 (33.2)	284 (38.2)		1543 (33.4)	311 (36.8)	
7–9.99 million	1220 (25.8)	141 (19.0)		1196 (25.9)	165 (19.5)	
≥ 10 million	860 (18.2)	104 (14.0)		862 (18.6)	102 (12.1)	
Unknown	542 (11.5)	85 (11.4)		524 (11.3)	103 (12.2)	
**CCHL scale**						
Low (first quartile)	1245 (26.4)	225 (30.2)	0.048	1184 (25.6)	286 (26.9)	< 0.001
Lower (second quartile)	1395 (29.5)	228 (30.6)		1376 (29.8)	247 (29.7)	
Higher (third quartile)	1147 (24.3)	166 (22.3)		1141 (24.7)	172 (24.0)	
High (fourth quartile)	935 (19.8)	125 (16.8)		621 (19.9)	139 (19.4)	

*CCHL scale* communicative and critical health literacy scale, *K6* Kessler 6 scale, *EPDS* Edinburgh postnatal depression scale.

^a^Using a Chi-squared test.

^b^Using Fisher’s exact test.

Table 3 shows the crude odd ratios (95% confidence intervals, CI) for ADs for each variable in pregnant woman using a univariate logistic regression model analysis. Pregnant women of an older age, higher education level, and higher CCHL had less ADs. Women in late pregnancy had less anxiety symptoms than those in early pregnancy. In comparisons with first-time pregnancies, pregnant women with one or two children had fewer ADs, while those with three children had more ADs. Pregnant women with complications (threatened premature delivery and fetal disorder or fetal growth restriction), a history of mental disease, separated and unmarried, housewives or students, those on leave, and those who were unemployed had more ADs. A history of hypertension was associated with more severe depressive syndrome.

**Table 3:** Relationships between anxiety and depressive symptoms for each variable in pregnant woman (n = 5466).

	Univariable logistic regression model analysis					
	K6 score ≥ 10		p value	EPDS score ≥ 13		p value
	CORs	95% CI		CORs	95% CI	
**Age group**						
≤ 19 yr	1.000			1.000		
20–29 yr	0.354	0.118–1.063	0.064	0.331	0.114–0.961	0.042
30–39 yr	0.259	0.086–0.776	0.016	0.213	0.074–0.616	0.004
40–49 yr	0.206	0.065–0.647	0.007	0.157	0.052–0.478	0.001
**Weeks of gestation**						
Early pregnancy (≤ 15 wk)	1.000			1.000		
Mid-pregnancy (16–27 wk)	0.888	0.701–1.123	0.573	0.910	0.726–1.140	0.411
Late pregnancy (≥ 28 wk)	0.806	0.651–0.998	0.048	0.837	0.682–1.026	0.086
**Number of children born**						
0	1.000			1.000		
1	0.836	0.694–1.006	0.058	0.698	0.581–0.838	< 0.001
2	0.636	0.455–0.889	0.008	0.663	0.488–0.903	0.009
≥ 3	1.667	1.030–2.699	0.038	1.918	1.230–2.992	0.004
Unknown	2.993	1.019–8.793	0.046	4.376	1.581–12.115	0.004
**Complications during pregnancy, yes vs no**						
Threatened premature delivery	1.330	1.019–1.736	0.036	1.809	1.428–2.292	< 0.001
Fetal disorder or fetal growth restriction	2.192	1.162–4.135	0.015	2.533	1.395–4.597	0.002
Placental malposition	0.593	0.286–1.229	0.160	0.888	0.492–1.603	0.694
Multiple pregnancy	0.744	0.356–1.554	0.431	1.590	0.922–2.741	0.095
Gestational hypertension	1.016	0.352–2.926	0.977	1.432	0.581–3.527	0.435
Gestational diabetes mellitus	0.658	0.421–1.028	0.066	0.977	0.677–1.411	0.901
Other	1.065	0.872–1.300	0.538	1.012	0.835–1.225	0.905
**Medical history, yes vs no**						
Miscarriage	0.899	0.736–1.099	0.299	0.864	0.713–1.046	0.134
Fatal death	0.956	0.505–1.810	0.889	1.097	0.615–1.956	0.754
Premature birth	0.885	0.493–1.590	0.683	0.831	0.471–1.464	0.521
Hypertension	0.745	0.264–2.106	0.579	2.550	1.281–5.073	0.008
Diabetes mellitus	0.865	0.258–2.897	0.814	1.735	0.691–4.356	0.241
Mental disease	5.077	3.868–6.655	< 0.001	3.758	2.859–4.941	< 0.001
Other	1.055	0.871–1.276	0.585	0.969	0.806–1.166	0.841
**Marital status**						
Married and live together	1.000			1.000		
Married and separated	1.292	0.842–1.981	0.241	1.878	1.298–2.717	< 0.001
Unmarried with a partner	2.249	1.268–3.989	0.006	3.101	1.837–5.234	< 0.001
Unmarried without a partner	3.049	1.734–5.361	< 0.001	1.881	1.024–3.455	0.042
Other	1.321	0.153–11.327	0.799	1.156	0.135–9.905	0.895
Unknown	2.202	0.708–6.848	0.173	3.467	1.256–9.567	0.016
**Education**						
Junior high school	1.000			1.000		
High school	0.507	0.313–0.822	0.006	0.501	0.319–0.786	0.003
College	0.405	0.253–0.648	< 0.001	0.364	0.234–0.566	< 0.001
University	0.369	0.233–0.584	< 0.001	0.280	0.182–0.432	< 0.001
Graduate school	0.302	0.175–0.523	< 0.001	0.182	0.106–0.312	< 0.001
Unknown	0.456	0.160–1.305	0.143	0.252	0.082–0.775	0.016
**Current employment status**						
Full-time	1.000			1.000		
Part-time	1.213	0.903–1.629	0.200	1.074	0.809–1.426	0.620
Housewife or student	1.531	1.268–1.848	< 0.001	1.265	1.056–1.516	0.011
On leave	1.418	1.141–1.762	0.002	1.414	1.155–1.731	< 0.001
Unemployed	3.137	1.864–5.279	< 0.001	4.123	2.552–6.663	< 0.001
Unknown	1.876	0.931–3.778	0.078	2.126	1.124–4.019	0.020
**Household income, yen**						
< 1 million	1.000			1.000		
1–3.99 million	0.943	0.401–2.216	0.893	0.671	0.319–1.407	0.291
4–6.99 million	0.698	0.301–1.618	0.401	0.421	0.203–0.873	0.020
7–9.99 million	0.446	0.191–1.042	0.062	0.288	0.138–0.603	< 0.001
≥ 10 million	0.466	0.198–1.098	0.081	0.247	0.117–0.522	< 0.001
Unknown	0.605	0.255–1.433	0.253	0.411	0.194–0.869	0.020
**CCHL scale**						
Low (first quartile)	1.000			1.000		
Lower (second quartile)	0.904	0.741–1.104	0.323	0.743	0.616–0.896	0.002
Higher (third quartile)	0.801	0.645–0.994	0.044	0.624	0.508–0.767	< 0.001
High (fourth quartile)	0.740	0.585–0.935	0.012	0.625	0.501–0.779	< 0.001

*CCHL scale* communicative and critical health literacy scale, *K6* Kessler 6 scale, *EPDS* Edinburgh postnatal depression scale, *CORs* crude odds ratios, *95% CI* 95% Confidence interval.

**Figure 1:**
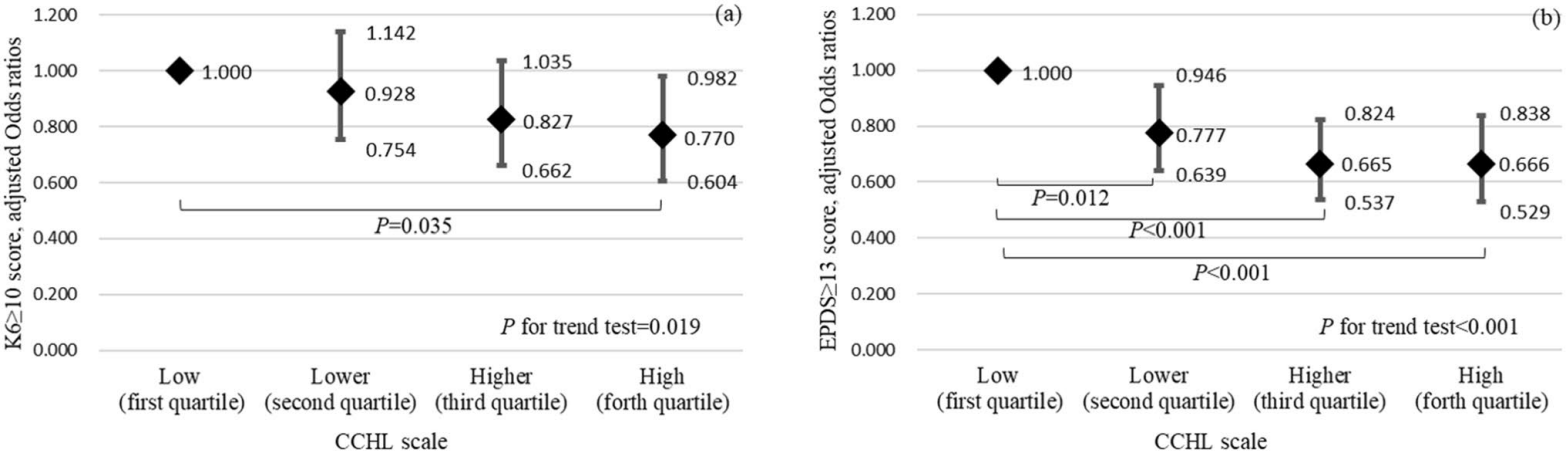
Anxiety (**a**) and depressive (**b**) symptoms in pregnant women in four CCHL groups. *CCHL* communicative and critical healthy literacy scale. Using a multivariable logistic regression model analysis with odds ratios (rhombus), 95% confidence intervals (bar), and *p* values, and the *p* for trend tests after adjustments for age, weeks of pregnancy, number of children born, complications during pregnancy, medical history, marital status, education, current employment status, and household income.

Spearman’s correlation coefficients for all potential confounding factor pairs were less than 0.597 (see Supplementary Table S1), and independent variables (22 items) multiplied 30 were less than 744 for a K6 score ≥ 10 and 844 for an EPDS score ≥ 13 in the multivariable logistic regression models. After adjustments for potential confounding factors, such as age, weeks of gestation, complications, medical history, the number of children, marital status, education level, employment status, and household income, the adjusted odds ratio (95% CI) for anxiety symptoms was significantly lower in the high CCHL group than in the low CCHL group [0.779 (0.604–0.982)]. The adjusted odds ratio (95% CI) for depressive symptoms was significantly lower in the lower CCHL group [0.777 (0.639–0.946)], higher CCHL group [0.665 (0.537–0.824)], and high CCHL group [0.666 (0.529–0.838)] than in the low CCHL group. A trend test showed significant trends in the adjusted odds ratio for anxiety symptoms (p for trend = 0.019) and depressive symptoms (*p* for trend < 0.001) (Fig. 1a,b). Supplementary Table S2 shows adjusted odds ratios (95% CI) for ADs to other variables.

## Discussion

In the present study, anxiety symptoms with a K6 score ≥ 10 in the high CCHL group and depressive symptoms with an EPDS score ≥ 13 in the lower, higher, and high CCHL groups showed significantly lower adjusted odds ratios than those in the lower CCHL group. The proportion of participants with ADs was lower in the low to high CCHL groups. To the best of our knowledge, this is the first study to report relationships between ADs and CCHL in pregnant women during the COVID-19 pandemic.

During the COVID-19 pandemic, 13.5% of pregnant women had anxiety symptoms with a K6 score ≥ 10 while 15.4% had depressive symptoms with an EPDS score ≥ 13 in the present study. These results were consistent with previous findings showing higher anxiety and depression in pregnant women after COVID-19 infection^4,7,8^, but were lower than those reported in other previous studies, with 7.5% of pregnant women having a K6 score (≥ 10)^16^ and 9.5% with a high EPDS score (≥ 13)^17^ before COVID-19 infection. This survey was conducted in September 2020. Although the cumulative incidence of patients with and deaths from COVID-19 was not high at that time in Japan, it was steadily increasing every day^18,19^. The high levels of ADs among pregnant women due to the COVID-19 pandemic were further validated in the present study.

On the other hand, approximately 60% or more of pregnant women in the present study were first-time mothers older than 30 years and in the last trimester of pregnancy at the time of the survey. Anxiety about pregnancy and childbirth was superimposed on COVID-19 infection, which was associated with more ADs. Further studies are warranted to develop strategies that improve ADs in pregnant women.

According to a basic 5-item CCHL showing that internal consistency was adequately high (Cronbach’s α = 0.86)^20^, for COVID-19, we used the CCHL and obtained Cronbach’s α = 0.78. Moreover, in consideration of some potential confounding factors associated with ADs, such as sociodemographic and socioeconomic factors^21^, we used a multivariable logistic model to analyze relationships with CCHL, and found that CCHL was still a dependent factor affecting ADs in pregnant women during the COVID-19 pandemic. As one upstream factor, health literacy is an individual’s ability to influence downstream health conditions^22^ or mortality rates^23^. In the present study, we measured CCHL for COVID-19. An insufficient knowledge of COVID-19 infection was suggested to be associated with ADs in pregnant women. Therefore, the present results are significant and reliable.

Vaccination and effective medication against COVID-19 are the only strategies for ADs caused by COVID-19. With the spread of vaccines worldwide, the Japan Society of Obstetrics and Gynecology has also recommended vaccines for pregnant women^24^. The widespread vaccination of pregnant women is expected to reduce ADs caused by COVID-19. However, the complete control of COVID-19 has not yet been achieved. Even if COVID-19 is eventually controlled, humans will undoubtedly encounter novel infections again in the future. The ability to collect, analyze, and use health information is a skill that is beneficial for everyone. In the present study, we focused on pregnant women and examined relationships between ADs and CCHL. As suggested by Paakkari and Okan^25^, CCHL may provide a solution that reduces ADs due to the impact of a novel infectious pandemic.

While not limited to pregnant women, many studies reported that the level of health literacy on COVID-19 was not only a cause of increased mental disorders, but also increased future anxiety^26,27^. Therefore, health literacy-based policy decisions and the provision of information as well as accurate knowledge and appropriate actions against novel infectious diseases are important^28,29^. However, the situation for pregnant women is more complex. In the present study, in addition to CCHL, weeks of gestation, the number of children, complications, a history of mental disease, marital status, education level, and employment status were related to ADs (Supplement Table S1). In consideration of these factors, detailed support appears to be needed while enhancing health literacy. For example, it is important to provide preconception and health education in routine prenatal classes^30^.

The limitations of the present study need to be addressed. (1) Since the present study had a cross-sectional design, causal relationships were not identified. (2) ADs were assessed using a self-reported questionnaire and not by a medical doctor; therefore, information bias was not avoided. (3) This was an online survey study; therefore, selection bias existed and the reproducibility of the results obtained cannot be evaluated. However, this was a large-scale survey with few missing values, respondents were from all prefectures in Japan, and the representative sample size was obtained. (4) Since there are no data on psychiatric comorbidities in pregnant women, a medical history of mental disease was adjusted for. (5) The careful interpretation of the present results is necessary for generalization to other populations due to the above limitations.

In conclusion, the present results suggest that CCHL had an impact on anxiety and depressive symptoms in pregnant women during the COVID-19 pandemic. Since COVID-19 has not yet been eradicated, psychological stress in pregnant women is likely to increase. Therefore, mental care and health literacy are considered to be equally important for pregnant women.

## Methods

### Study design and subjects

A cross-sectional study was conducted using an online survey for pregnant women in Japan between September 1st and 30th, 2020, through leaflets delivered to medical facilities and placed on social networking sites, such as Facebook, Twitter, and Line. The Japan Society of Obstetrics and Gynecology (https://www.jsog.or.jp/), Yokohama City University School of Medicine (https://www.yokohama-cu.ac.jp/academics/med/index.html), Pregnant Women Health Initiative (https://pw-hi.jp/), and Registration for COVID-19 complicated pregnancy in Japan (https://www.med.kobe-u.ac.jp/cmv/covid/) were used. Participants older than 20 years or married minors 16–19 years old, who are considered to be adults under Japanese Civil Law at the time of the survey (https://elaws.e-gov.go.jp/document?lawid=129AC0000000089) and have sufficient judgment under Ethical Guidelines Guidance in Japan (https://www.mhlw.go.jp/content/000946358.pdf), were recruited in the present study. Exclusion criteria were unmarried women between the ages of 16 and 19 years old or women younger than 16 years old, women whose gestational weeks and expected date of delivery did not match, or one of the CCHL, K6, and EPDS items was defective and inadequate. Informed consent to participate in this study was obtained from potential participants prior to answering the questionnaire and one of the most secure online questionnaire sites, “SurveyMonkey™” was used. At the beginning of the survey, written informed consent on WEB site stated that the survey was voluntary and that even if potential participants began to respond, it may be stopped prematurely. In addition, the agreement that pressing the submit button before and after answering the questions was considered the final agreement was a requirement to proceed to the actual research website. Information sent on the internet was encrypted and converted into data through a secure server without individual information. Online survey questions included the characteristics and socioeconomic status of pregnant women, such as age, weeks of gestation, number of children, complications during pregnancy, medical history, marital status, education level, employment status, and household income.

### Assessments of anxiety and depressive symptoms as well as health literacy

K6^31^ and EDPS^32^ with a 5-point Likert scale were used to assess ADs in pregnant women. In the present study, Cronbach’s coefficient alpha of K6 and EPDS were 0.860 and 0.875, respectively. A cut-off value of a K6 score of 10 indicated anxiety symptoms in pregnant women, while an EPDS score of 13 indicated depressive symptoms.

The CCHL levels of pregnant women were assessed using a basic 5-item, and a 5-point Likert scale of CCHL^20,33^ for COVID-19. The 5 items consist of the following: (1) the ability to gather information on COVID-19 from various sources; (2) the ability to select information necessary for oneself (on childbirth and postpartum) from a large amount of information on COVID-19; (3) an understanding of information on COVID-19 and the ability to convey it to others; (4) the ability to judge the reliability of information on COVID-19; and (5) the ability to decide on plans and actions to prevent infection based on information on COVID-19. In the present study, Cronbach’s coefficient alpha of CCHL was 0.783.

### Statistical analysis

Sociodemographic factors, pregnancy-related factors, medical history, anxiety symptoms (K6), depressive symptoms (EPDS), and CCHL were assessed using descriptive statistics. To analyze the trend relationship with ADs, CCHL scores were divided into four groups: low (first quartile), lower (second quartile), higher (third quartile), and high (fourth quartile) levels, based on the quartile of the distribution of CCHL.

The proportions of anxiety symptoms (K6 score ≥ 10) and depressive symptoms (EPDS score ≥ 13) for each variable were analyzed using the Chi-squared test or Fisher’s exact test when the expected number of zero cells was 20% or more. A univariable logistic regression model was used to examine the relationship between each variable and ADs. To analyze the relationship between the four CCHL groups of pregnant women and ADs, multivariable logistic regression models and trend tests were conducted after adjustments for all potential confounding factors, such as age, weeks of pregnancy, number of children born, complications during pregnancy, medical history, marital status, education, current employment status, and household income. The multicollinearity of the input variables was confirmed with Spearman’s correlation coefficient less than 0.9. The multivariate overfitting was checked by multiplying the number of input variables by 30 and below the number of K6 scores ≥ 10 or EPDS scores ≥ 13.

All statistical analyses were performed using a two-tailed test and an assumed type I error rate of 0.05. Statistical analyses were performed using IBM SPSS Statistics 27 for Windows (IBM Japan, Tokyo, Japan).

### Ethical approval and informed consent

The present study was approved by the Institutional Ethics Committee of Yokohama City University (B 200800046), and all methods were performed in accordance with the Declaration of Helsinki, relevant guidelines, and regulations. Consent to participate in the present study was obtained by confirmation from participants at the start of questionnaire responses.

## Supplementary Information

Supplementary Tables.

## Data Availability

The datasets used and/or analyzed during the current study available on the following web site of the Department of Obstetrics and Gynecology, Yokohama City University Graduate School of Medicine, Japan. The datasets on CCHC in pregnant women is available at https://pw-hi.jp/.
