# Supplementary material for: Impact of health literacy on anxiety and depressive symptoms in pregnant women in Japan during the COVID-19 pandemic
Source: Sci Rep. 2022 Aug 18;12:14042. doi: 10.1038/s41598-022-18405-3 (PMC9386675; doi:10.1038/s41598-022-18405-3)
Supplement: Supplementary file 1 — Supplementary Tables. [file 41598_2022_18405_MOESM1_ESM.docx]

Table S1 Spearman’s correlation coefficients on the variables in pregnant woman (n=5466)

|  | Variables | 1 | 2 | 3 | 4 | 5 | 6 | 7 | 8 | 9 | 10 | 11 | 12 | 13 | 14 | 15 | 16 | 17 | 18 | 19 | 20 | 21 | 22 |
| --- | --- | --- | --- | --- | --- | --- | --- | --- | --- | --- | --- | --- | --- | --- | --- | --- | --- | --- | --- | --- | --- | --- | --- |
| 1 | CCHL scale | 1.000 | -0.002 | 0.056 | -0.007 | -0.020 | 0.012 | 0.023 | 0.015 | 0.005 | 0.004 | 0.034 | -0.034 | 0.051 | 0.013 | 0.015 | -0.003 | 0.001 | -0.004 | -0.029 | -0.015 | -0.002 | 0.010 |
| 2 | Age group |  | 1.000 | -0.032 | 0.200 | 0.010 | -0.014 | 0.031 | 0.006 | 0.049 | 0.073 | 0.023 | -0.018 | 0.117 | -0.028 | 0.123 | 0.158 | 0.065 | 0.051 | 0.048 | 0.032 | -0.006 | 0.000 |
| 3 | Weeks of gestation |  |  | 1.000 | -0.035 | 0.138 | 0.047 | 0.034 | -0.014 | 0.030 | 0.082 | -0.027 | -0.036 | -0.008 | 0.184 | -0.016 | -0.048 | 0.011 | -0.015 | -0.026 | 0.019 | 0.003 | -0.016 |
| 4 | Number of children born |  |  |  | 1.000 | 0.057 | 0.003 | 0.002 | -0.010 | 0.047 | -0.006 | -0.029 | -0.058 | -0.106 | 0.081 | -0.037 | 0.117 | 0.099 | 0.182 | 0.000 | 0.028 | -0.008 | -0.069 |
| 5 | Threatened premature delivery |  |  |  |  | 1.000 | 0.007 | 0.036 | 0.047 | 0.054 | 0.031 | -0.092 | -0.010 | -0.077 | 0.113 | 0.008 | 0.044 | 0.019 | 0.113 | -0.016 | -0.020 | 0.023 | -0.005 |
| 6 | Fetal disorder or fetal growth restriction |  |  |  |  |  | 1.000 | 0.017 | -0.012 | 0.019 | 0.008 | -0.040 | 0.001 | -0.023 | 0.002 | -0.013 | 0.005 | 0.019 | -0.014 | -0.008 | -0.007 | 0.017 | 0.013 |
| 7 | Placental malposition |  |  |  |  |  |  | 1.000 | -0.004 | 0.029 | 0.000 | -0.046 | -0.019 | 0.010 | 0.026 | 0.007 | 0.053 | 0.030 | -0.008 | 0.006 | -0.009 | -0.007 | 0.019 |
| 8 | Multiple pregnancy |  |  |  |  |  |  |  | 1.000 | 0.013 | 0.014 | -0.051 | -0.015 | 0.003 | 0.034 | 0.013 | 0.021 | -0.002 | -0.017 | 0.009 | -0.008 | -0.025 | 0.015 |
| 9 | Gestational hypertension |  |  |  |  |  |  |  |  | 1.000 | 0.085 | -0.034 | -0.006 | -0.027 | -0.007 | -0.007 | -0.010 | -0.009 | 0.026 | 0.206 | 0.032 | -0.015 | -0.018 |
| 10 | Gestational diabetes mellitus |  |  |  |  |  |  |  |  |  | 1.000 | -0.083 | 0.009 | -0.006 | 0.021 | 0.020 | 0.030 | 0.026 | 0.030 | 0.026 | 0.107 | -0.004 | -0.037 |
| 11 | Other (complications) |  |  |  |  |  |  |  |  |  |  | 1.000 | 0.027 | 0.038 | -0.034 | 0.011 | -0.017 | 0.000 | -0.006 | -0.004 | -0.010 | 0.005 | 0.597 |
| 12 | Marital status |  |  |  |  |  |  |  |  |  |  |  | 1.000 | -0.071 | 0.009 | -0.020 | -0.025 | -0.017 | -0.005 | 0.028 | 0.044 | 0.032 | 0.010 |
| 13 | Education |  |  |  |  |  |  |  |  |  |  |  |  | 1.000 | -0.148 | 0.263 | -0.011 | -0.016 | -0.033 | -0.026 | -0.022 | -0.038 | 0.026 |
| 14 | Current employment status |  |  |  |  |  |  |  |  |  |  |  |  |  | 1.000 | -0.147 | 0.021 | 0.018 | 0.054 | 0.008 | 0.016 | 0.063 | -0.026 |
| 15 | Household income |  |  |  |  |  |  |  |  |  |  |  |  |  |  | 1.000 | 0.023 | -0.010 | -0.011 | -0.010 | -0.018 | -0.052 | 0.014 |
| 16 | Miscarriage |  |  |  |  |  |  |  |  |  |  |  |  |  |  |  | 1.000 | 0.067 | 0.072 | 0.009 | 0.008 | -0.004 | -0.119 |
| 17 | Fatal death |  |  |  |  |  |  |  |  |  |  |  |  |  |  |  |  | 1.000 | 0.036 | 0.007 | 0.036 | -0.004 | -0.037 |
| 18 | Premature birth |  |  |  |  |  |  |  |  |  |  |  |  |  |  |  |  |  | 1.000 | -0.012 | 0.049 | -0.004 | -0.047 |
| 19 | Hypertension |  |  |  |  |  |  |  |  |  |  |  |  |  |  |  |  |  |  | 1.000 | 0.060 | 0.015 | -0.031 |
| 20 | Diabetes mellitus |  |  |  |  |  |  |  |  |  |  |  |  |  |  |  |  |  |  |  | 1.000 | -0.001 | -0.027 |
| 21 | Mental disease |  |  |  |  |  |  |  |  |  |  |  |  |  |  |  |  |  |  |  |  | 1.000 | -0.045 |
| 22 | Other (medical history) |  |  |  |  |  |  |  |  |  |  |  |  |  |  |  |  |  |  |  |  |  | 1.000 |

CCHL scale: Communicative and critical health literacy scale

Table S2 Relationship between anxiety or depressive symptoms and CCHL in pregnant woman (n=5466)

|  | Multivariable logistic regression model analysis^a^ | | | | | | |  |
| --- | --- | --- | --- | --- | --- | --- | --- | --- |
|  | K6 score ≥10 | |  |  | EPDS score ≥13 | |  | |
|  | AORs | 95%CI | p value |  | AORs | 95%CI | p value | |
| Age group |  |  |  |  |  |  |  | |
| ≤19 yr | 1.000 |  |  |  | 1.000 |  |  | |
| 20-29 yr | 0.764 | 0.215-2.713 | 0.677 |  | 0.731 | 0.220-2.434 | 0.610 | |
| 30-39 yr | 0.618 | 0.174-2.201 | 0.458 |  | 0.544 | 0.163-1.817 | 0.322 | |
| 40-49 yr | 0.488 | 0.131-1.822 | 0.286 |  | 0.369 | 0.105-1.297 | 0.120 | |
| Weeks of gestation |  |  |  |  |  |  |  | |
| Early pregnancy (≤15 wk) | 1.000 |  |  |  | 1.000 |  |  | |
| Mid-pregnancy (16-27 wk) | 0.844 | 0.660-1.079 | 0.176 |  | 0.846 | 0.667-1.072 | 0.166 | |
| Late pregnancy (≥28 wk) | 0.720 | 0.572-0.905 | 0.005 |  | 0.702 | 0.562-0.877 | 0.002 | |
| Number of children born |  |  |  |  |  |  |  | |
| 0 | 1.000 |  |  |  | 1.000 |  |  | |
| 1 | 0.856 | 0.702-1.045 | 0.123 |  | 0.713 | 0.586-0.868 | <0.001 | |
| 2 | 0.640 | 0.449-0.911 | 0.013 |  | 0.666 | 0.480-0.925 | 0.015 | |
| ≥3 | 1.449 | 0.860-2.442 | 0.163 |  | 1.566 | 1.008-2.677 | 0.046 | |
| Unknown | 2.989 | 0.892-10.109 | 0.076 |  | 4.983 | 1.566-15.855 | 0.007 | |
| Complications during pregnancy, yes vs no |  |  |  |  |  |  |  | |
| Threatened premature delivery | 1.362 | 1.019-1.821 | 0.037 |  | 1.897 | 1.461-2.463 | <0.001 | |
| Fetal disorder or fetal growth restriction | 2.302 | 1.184-4.475 | 0.014 |  | 2.636 | 1.404-4.949 | 0.003 | |
| Placental malposition | 0.618 | 0.291-1.313 | 0.211 |  | 0.965 | 0.515-1.808 | 0.911 | |
| Multiple pregnancy | 0.795 | 0.375-1.685 | 0.550 |  | 1.744 | 0.989-3.075 | 0.055 | |
| Gestational hypertension | 1.411 | 0.458-4.348 | 0.548 |  | 1.311 | 0.495-3.467 | 0.586 | |
| Gestational diabetes mellitus | 0.731 | 0.460-1.162 | 0.186 |  | 1.102 | 0.746-1.627 | 0.626 | |
| Other | 1.050 | 0.810-1.360 | 0.713 |  | 1.179 | 0.918-1.515 | 0.197 | |
| Medical history, yes vs no |  |  |  |  |  |  |  | |
| Miscarriage | 0.974 | 0.787-1.205 | 0.809 |  | 0.932 | 0.758-1.145 | 0.500 | |
| Fatal death | 0.968 | 0.476-0.971 | 0.929 |  | 0.983 | 0.505-1.915 | 0.961 | |
| Premature birth | 0.857 | 0.461-1.592 | 0.626 |  | 0.748 | 0.408-1.372 | 0.349 | |
| Hypertension | 0.552 | 0.185-1.650 | 0.287 |  | 1.929 | 0.898-4.141 | 0.092 | |
| Diabetes mellitus | 1.022 | 0.292-3.576 | 0.973 |  | 1.782 | 0.659-4.819 | 0.255 | |
| Mental disease | 4.653 | 3.305-6175 | <0.001 |  | 3.498 | 2.619-4.673 | <0.001 | |
| Other | 1.074 | 0.838-1.375 | 0.571 |  | 0.925 | 0.728-1.177 | 0.526 | |
| Marital status |  |  |  |  |  |  |  | |
| Married and live together | 1.000 |  |  |  | 1.000 |  |  | |
| Married and separated | 1.121 | 0.715-1.757 | 0.619 |  | 1.644 | 1.125-2.462 | 0.011 | |
| Unmarried with a partner | 1.818 | 0.986-3.352 | 0.055 |  | 1.953 | 1.099-3.471 | 0.023 | |
| Unmarried without a partner | 2.162 | 1.171-3.992 | 0.014 |  | 0.997 | 0.513-1.938 | 0.994 | |
| Other | 1.367 | 0.150-12.417 | 0.781 |  | 1.153 | 0.120-11.112 | 0.902 | |
| Unknown | 1.756 | 0.512-6.030 | 0.371 |  | 2.846 | 0.917-8.833 | 0.070 | |
| Education |  |  |  |  |  |  |  | |
| Junior high school | 1.000 |  |  |  | 1.000 |  |  | |
| High school | 0.659 | 0.384-1.133 | 0.131 |  | 0.666 | 0.401-1.107 | 0.177 | |
| College | 0.636 | 0.372-1.088 | 0.098 |  | 0.577 | 0.348-0.955 | 0.033 | |
| University | 0.642 | 0.376-1.096 | 0.104 |  | 0.505 | 0.305-0.837 | 0.008 | |
| Graduate school | 0.604 | 0.324-1.125 | 0.112 |  | 0.373 | 0.203-0.687 | 0.002 | |
| Unknown | 0.704 | 0.230-2.160 | 0.540 |  | 0.367 | 0.112-1.208 | 0.099 | |
| Current employment status |  |  |  |  |  |  |  | |
| Full-time | 1.000 |  |  |  | 1.000 |  |  | |
| Part-time | 1.007 | 0.733-1.384 | 0.966 |  | 0.792 | 0.583-1.077 | 0.137 | |
| Housewife or student | 1.319 | 1.069-1.629 | 0.010 |  | 0.982 | 0.801-1.203 | 0.860 | |
| On leave | 1.378 | 1.092-1.738 | 0.007 |  | 1.246 | 1.001-1.550 | 0.049 | |
| Unemployed | 2.180 | 1.254-3.789 | 0.006 |  | 2.476 | 1.471-4.166 | <0.001 | |
| Unknown | 1.806 | 0.846-3.857 | 0.126 |  | 1.795 | 0.888-3.631 | 0.103 | |
| Household income, yen |  |  |  |  |  |  |  | |
| <1 million | 1.000 |  |  |  | 1.000 |  |  | |
| 1-3.99 million | 1.208 | 0.482-3.027 | 0.688 |  | 0.785 | 0.347-1.744 | 0.560 | |
| 4-6.99 million | 1.064 | 0.429-2.643 | 0.893 |  | 0.593 | 0.265-1.328 | 0.204 | |
| 7-9.99 million | 0.745 | 0.296-1.873 | 0.531 |  | 0.441 | 0.195-1.001 | 0.050 | |
| ≥10 million | 0.845 | 0.332-2.150 | 0.723 |  | 0.410 | 0.178-0.943 | 0.036 | |
| Unknown | 0.885 | 0.349-2.246 | 0.798 |  | 0.537 | 0.355-1.228 | 0.141 | |
| CCHL scale |  |  |  |  |  |  |  | |
| Low (first quartile) | 1.000 |  |  |  | 1.000 |  |  | |
| Lower (second quartile) | 0.928 | 0.754-1.142 | 0.480 |  | 0.777 | 0.639-0.946 | 0.012 | |
| Higher (third quartile) | 0.827 | 0.662-1.035 | 0.097 |  | 0.665 | 0.537-0.824 | <0.001 | |
| High (fourth quartile) | 0.770 | 0.604-0.982 | 0.035 |  | 0.666 | 0.529-0.838 | <0.001 | |

CCHL scale: Communicative and critical health literacy scale, K6: Kessler 6 scale, EPDS: Edinburgh postnatal depression scale, AORs: Adjusted odds ratios, 95%CI: 95% Confidence interval.

a: All variables were entered into the multivariable logistic regression model.
